# Development and Analytical Validation of a Laboratory‐Customized TaqMan‐MGB Probe Method for *CYP2C19* Genotyping

**DOI:** 10.1002/jcla.70296

**Published:** 2026-07-01

**Authors:** Yaqun Liu, Lianghui Chen, Xuanyi Zheng, Peikui Yang, Yicun Chen, Chengsong Xie, Zhenxia Zhang, Yuzhong Zheng

**Affiliations:** ^1^ Guangdong Key Laboratory of Functional Substances and Health Products From Medicinal Edible Resources Hanshan Normal University Chaozhou China; ^2^ Shantou University Medical College Shantou China; ^3^ Guangdong Taiantang Pharmaceutical Co. Ltd. Shantou China; ^4^ Industrial College of Biomedicine and Health Industry Youjiang Medical University for Nationalities Baise China; ^5^ Second Clinical Medical College Southern Medical University Guangzhou China

**Keywords:** analytical validation, *CYP2C19*, polymorphism, TaqMan‐MGB

## Abstract

**Background:**

Genetic polymorphisms in *CYP2C19* influence the metabolism of multiple clinically important drugs. Although TaqMan‐based genotyping of CYP2C19*2 and CYP2C19*17 is well established, open and locally implementable workflows remain useful for laboratory validation and application. This study aimed to develop and analytically validate a laboratory‐customized, open‐sequence TaqMan‐MGB assay for detecting CYP2C19*2 (rs4244285, NM_000769.4:c.681G > A) and CYP2C19*17 (rs12248560, NM_000769.4:c.‐806C > T).

**Methods:**

Specific primers and allele‐specific TaqMan‐MGB probes were designed. The assay was evaluated for primer/probe performance, specificity, analytical sensitivity, candidate limit of detection (LOD), and repeatability. Preliminary clinical concordance was assessed using 20 dried blood spot (DBS) samples, with Sanger sequencing as the reference.

**Results:**

The assay discriminated wild‐type, heterozygous, and homozygous mutant genotypes for CYP2C19*2 and wild‐type and heterozygous genotypes for CYP2C19*17 in tested clinical samples. Using plasmid templates, candidate LODs were 1.17 × 10^2^ copies/μL for CYP2C19*2 and 0.94 × 10^3^ copies/μL for CYP2C19*17. Repeatability testing with plasmid templates and representative DBS‐derived clinical genomic DNA showed coefficients of variation below 10%. All 20 DBS samples were concordant with Sanger sequencing, corresponding to 100% observed sample‐level concordance and an exact 95% confidence interval of 83.2%–100.0%.

**Conclusion:**

The customized TaqMan‐MGB assay showed acceptable preliminary analytical specificity, sensitivity, and repeatability. Its value lies in the laboratory‐customized, open‐sequence design and DBS‐compatible workflow. Because the clinical set was limited and lacked the CYP2C19*17 TT genotype, the findings should be regarded as preliminary. Larger studies including all relevant genotypes, additional *CYP2C19* alleles, and diverse populations are needed before routine clinical implementation or comprehensive *CYP2C19* phenotype assignment.

## Graphical abstract

A laboratory‐customized, open‐sequence TaqMan‐MGB probe assay was developed for detecting CYP2C19*2 and CYP2C19*17 polymorphisms using dried blood spot‐derived DNA. The assay demonstrated allele‐specific discrimination, candidate LODs of 1.17 × 10^2^ copies/μL for CYP2C19*2 and 0.94 × 10^3^ copies/μL for CYP2C19*17, and repeatability with CVs below 10%. In a preliminary clinical evaluation of 20 DBS samples, genotyping results were fully concordant with Sanger sequencing, supporting further large‐scale validation of this customizable pharmacogenetic workflow.

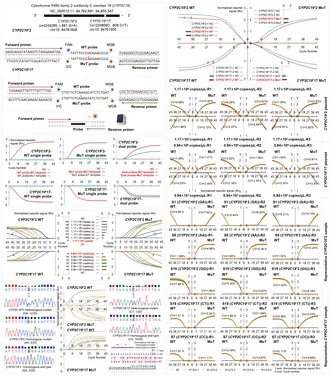

## 1 Introduction

The *CYP2C19* gene encodes cytochrome P450 2C19, a crucial enzyme involved in the metabolism of various medications, including proton pump inhibitors, antidepressants, and antiplatelet drugs [1]. Genetic polymorphisms within the *CYP2C19* gene significantly affect enzyme activity, leading to interindividual differences in drug response and efficacy [2]. Among the numerous polymorphisms identified in the *CYP2C19* gene, CYP2C19*2 and CYP2C19*17 are particularly noteworthy because of their high prevalence and clinical relevance. CYP2C19*2 is a loss‐of‐function allele caused by a single‐nucleotide polymorphism (681G > A), resulting in a splicing defect and the production of a nonfunctional enzyme. This polymorphism reduces *CYP2C19* function and may contribute to intermediate or poor metabolizer phenotypes depending on the diplotype, thereby affecting drug metabolism and potentially increasing the risk of adverse drug reactions or therapeutic failure [3]. Conversely, CYP2C19*17 is an increased‐function allele characterized by the −806C > T polymorphism and may contribute to rapid or ultrarapid metabolizer phenotypes depending on the diplotype. For certain drugs, individuals carrying increased‐function *CYP2C19* alleles may have altered plasma concentrations, and genotype‐informed dose adjustment may be considered according to drug‐specific guidelines and clinical context [4]. This study focused on these two polymorphic loci because of their significant implications in personalized medicine.

Over the years, various methodologies have been developed to identify *CYP2C19* genetic polymorphisms, including PCR‐RFLP [5], Sanger sequencing [6], real‐time quantitative PCR [7], gene chip hybrid analysis [8], and high‐throughput sequencing technologies [9]. While each of these techniques offers distinct advantages, they also have inherent limitations. For example, PCR‐RFLP is straightforward and cost‐effective but suffers from low efficiency, whereas gene chip hybrid analysis and high‐throughput sequencing are highly efficient yet associated with elevated costs. Among these methods, TaqMan minor groove binder (TaqMan‐MGB) probe technology has emerged as a widely used approach because of its high specificity, sensitivity, and ease of operation. This technique uses a specially designed fluorescent probe labeled at the 5′ end with a reporter dye and conjugated at the 3′ end with a minor groove binder (MGB) moiety. MGB enhances the probe's binding stability and specificity to the target sequence [10, 11]. During amplification, the probe hybridizes with the target DNA, and its degradation by the 5′ → 3′ exonuclease activity of DNA polymerase releases a fluorescent signal, enabling real‐time monitoring of gene amplification. TaqMan‐MGB probe technology has been extensively applied in the detection of gene polymorphisms, including *CYSLTR1* T927C, *GSDMB* G1199A [12], *MTHFR* C677T and A1298C [13], and *CSPG4P12* [14], providing a robust and reliable tool for genetic analysis in pharmacogenomics and related fields. TaqMan‐based assays for CYP2C19*2 and CYP2C19*17 have been reported in previous pharmacogenetic studies and are available as commercial genotyping assays, such as the TaqMan OpenArray Pharmacogenomics (PGx) Express Panel (Thermo Fisher Scientific, https://www.thermofisher.cn/order/catalog/product/4488847), Human *CYP2C19* Gene Polymorphism Detection Kit (Fuyuanbio, http://www.fuyuanbio.com/fuyuanbio/products/19087371.html), and other proprietary or closed‐kit platforms. Therefore, the development of another TaqMan assay for these two alleles should be justified by its specific assay design, workflow characteristics, and validation context rather than by the detection principle alone.

Although TaqMan‐based assays for *CYP2C19* genotyping are commercially available and have been widely used, the present study was designed to establish and analytically validate a laboratory‐customized TaqMan‐MGB assay with disclosed primer and probe sequences. The potential value of this approach lies in its flexibility, local implementability, compatibility with dried blood spot (DBS)‐derived DNA, and suitability for laboratories seeking a customizable alternative to closed commercial kits. Therefore, the contribution of this study is not the TaqMan‐MGB principle itself, but the combination of disclosed primer/probe design, empirical primer‐set optimization, adjusted FAM/VIC probe ratio for allele discrimination, Chelex‐100‐based DNA extraction from DBS, and analytical validation under the described conditions. In this study, we designed and optimized specific primers and probes for two clinically relevant *CYP2C19* polymorphic sites, CYP2C19*2 and CYP2C19*17, and evaluated the analytical specificity, sensitivity, repeatability, and preliminary clinical concordance of the assay using DBS samples.

## 2 Materials and Methods

### 2.1 Design and Synthesis of Primers and TaqMan‐MGB Probes

The primers and TaqMan‐MGB probes were designed via Primer Express v3.0 and the National Center for Biotechnology Information (NCBI) Primer‐BLAST (http://www.ncbi.nlm.nih.gov/tools/primer‐blast) [15]. For each locus, two primer combinations were initially designed around the target SNP region, while allele‐specific MGB probes were designed to place the polymorphic nucleotide within the probe‐binding region to improve allele discrimination. Candidate primers were screened in silico to reduce nonspecific amplification against the human reference genome. All oligonucleotides were synthesized by General Biosystems (Anhui) Co. Ltd. The specific sequences are detailed in Table 1.

**TABLE 1:** Primer and probe sequences for the CYP2C19*2 and CYP2C19*17 loci.

Locus	Primer/Probe	Sequence (5′ → 3′)*	Length (nt)
CYP2C19*2 (rs4244285, NM_000769.4:c.681G > A)*	F1	AAGCAGGTATAAGTCTAGGAAATGA	25
	F2	AAGCAGGTATAAGTCTAGGAAATGA	25
	R1	AGTCCCGAGGGTTGTTGA	18
	R2	ATAAAGTCCCGAGGGTTGTT	20
	Wild‐type Probe	FAM‐TATTTCCCGGGAACCCA‐NFQ‐MGB	17
	Mutant Probe	VIC‐TATTTCCCAGGAACCCA‐NFQ‐MGB	17
CYP2C19*17 (rs12248560, NM_000769.4:c.‐806C > T)*	F1	TTTGGAAGTTGTTTTGTTTTGC	22
	F2	TGGAAGTTGTTTTGTTTTGC	20
	R1	TGATGCCCATCGTGGC	16
	R2	GGGAGACCCTGGGAGAA	17
	Wild‐type Probe	FAM‐CTGTTCTCAAAGCATCTCTGAT‐NFQ‐MGB	22
	Mutant Probe	VIC‐CTGTTCTCAAAGTATCTCTGAT‐NFQ‐MGB	22

Abbreviations: FAM, 6‐carboxyfluorescein; MGB, minor groove binder; NFQ, non‐fluorescent quencher; VIC, proprietary fluorescent dye.

* Probes were labeled with FAM or VIC at the 5′ end and an MGB non‐fluorescent quencher at the 3′ end.

### 2.2 DNA Extraction and TaqMan‐MGB PCR Amplification Protocol

A 5% Chelex‐100 suspension was prepared using Chelex 100 Resin (Bio‐Rad, Cat. No. 1421253), in nuclease‐free ddH_2_O and stored at 4°C. Because Chelex‐100 is a resin suspension rather than a true solution, it was thoroughly vortexed and resuspended before each extraction. The Chelex‐100 suspension recovered after sample extraction was not reused. For each DBS, three 3‐mm DBS discs were routinely used when the blood spots were sufficiently saturated and homogeneous. For DBS samples with weak saturation, small spot size, or uneven blood distribution, up to five discs were used to obtain sufficient DNA for PCR amplification and downstream Sanger sequencing confirmation. Each tube was filled with 1 mL of ddH_2_O, vortexed for 1 min, and incubated at room temperature for 1 h. After centrifugation at 13,000 rpm for 3 min, the supernatant was discarded. Two hundred microliters of 5% Chelex‐100 suspension was subsequently added, vortexed briefly for 10 s, and heated at 56°C for 2 h with gentle mixing every 30 min. The samples were then heated at 98°C for 10 min and centrifuged, and the supernatant containing the extracted DNA was collected and stored at −20°C. Because the Chelex‐100 method yields crude DBS‐derived DNA extracts, DNA concentration and purity were not systematically measured for all clinical samples in the original workflow. The extracts were directly used for TaqMan‐MGB PCR amplification, and sample suitability was assessed primarily based on successful real‐time PCR amplification curves and subsequent Sanger sequencing confirmation. TaqMan‐MGB PCR amplification was performed in a 25 μL reaction volume containing 12.5 μL of Probe qPCR Super PreMix (Vazyme qv114), 1 μL each of forward and reverse primers (10 μM), 0.5 μL of the wild‐type TaqMan‐MGB probe (10 μM), 2.5 μL of the mutant TaqMan‐MGB probe (10 μM), 5 μL of template DNA, and ddH_2_O to adjust the final volume. The unequal probe volumes were determined empirically during assay optimization to balance the FAM and VIC fluorescence signals and improve allele discrimination under the tested reaction conditions. The thermal cycling conditions consisted of initial denaturation at 95°C for 30 s, followed by 45 cycles of 95°C for 5 s, 60°C for 30 s, and 72°C for 20 s, with a final extension at 72°C for 5 min. This cycling program was based on the manufacturer's recommended protocol for the Probe qPCR Super PreMix, Vazyme qv114 (https://bio.vazyme.com/companyfile/1713.html). Fluorescence signals were collected during each amplification cycle, and all amplification curves were plotted as normalized reporter signal (Rn) versus cycle number. PCR amplicons covering the target SNPs were analyzed by 1.5% agarose gel electrophoresis and subjected to Sanger sequencing by Sangon Biotech (Shanghai) Co. Ltd.

### 2.3 Validation of the Specificity, Repeatability, and Sensitivity of the TaqMan‐MGB Assay

The TaqMan‐MGB assay was validated through specificity, repeatability, and sensitivity experiments to ensure its reliability and robustness. For the specificity analysis, plasmid DNA templates representing wild‐type and mutant alleles at two target loci were used. The assay was evaluated via both single‐probe and dual‐probe configurations to confirm the specificity of the selected primers and probes. Repeatability was evaluated using both plasmid DNA templates and DBS‐derived clinical genomic DNA under the same replicate‐testing scheme. For each template type, three independent experimental runs were performed on separate occasions and designated R1, R2, and R3. In each run, a new PCR reaction mixture was prepared, and each tested template was analyzed in five parallel wells, namely R1‐1 to R1‐5, R2‐1 to R2‐5, and R3‐1 to R3‐5, resulting in 15 replicate reactions for each template condition. For plasmid DNA testing, high‐ and low‐concentration templates were evaluated for each locus. The concentrations were 1.17 × 10^8^ and 1.17 × 10^2^ copies/μL for CYP2C19*2, and 0.94 × 10^8^ and 0.94 × 10^3^ copies/μL for CYP2C19*17. In each independent run, plasmid dilutions were freshly prepared before PCR amplification. For clinical‐matrix repeatability testing, DBS‐derived genomic DNA extracted using the Chelex‐100 method was analyzed using representative clinical samples with genotypes confirmed by Sanger sequencing. Five clinical DNA extracts were selected: one CYP2C19*2 heterozygous sample, GA; one CYP2C19*2 homozygous mutant sample, AA; one CYP2C19*2 wild‐type sample, GG; one CYP2C19*17 heterozygous sample, CT; and one CYP2C19*17 wild‐type sample, CC. The consistency of the results and the coefficient of variation (CV) were analyzed to assess the repeatability of the assay. CV values were calculated from endpoint ΔRn fluorescence signals at the final amplification cycle. For each assay condition, endpoint ΔRn values from replicate reactions were used to calculate CV as follows: CV (%) = standard deviation of endpoint ΔRn/mean endpoint ΔRn × 100 [16, 17]. The analytical sensitivity of the assay was assessed by performing gradient dilutions of plasmid DNA templates, followed by amplification. For the CYP2C19*2 locus, the DNA concentrations ranged from 1.17 × 10^8^ to 1.17 × 10^2^ copies/μL, whereas for the CYP2C19*17 locus, the DNA concentrations ranged from 0.94 × 10^8^ to 0.94 × 10^3^ copies/μL. The lowest concentration showing positive amplification in the dilution series was considered the candidate limit of detection (candidate LOD). The candidate LOD concentrations were then further tested in three independent runs, with five parallel reactions per run. Both wild‐type and mutant plasmid templates were tested, resulting in 15 replicate reactions for the wild‐type template and 15 replicate reactions for the mutant template at each locus. A reaction was considered positive when the amplification curve in the corresponding allele‐specific fluorescence channel crossed the preset threshold within 45 cycles and the no‐template control remained negative. Because the number of replicates and dilution levels was limited, these values were defined as candidate LODs rather than formal analytical LODs. ddH_2_O was included as the negative control in all experiments to exclude contamination and validate the accuracy of the assay.

### 2.4 Clinical Sample Collection and Preliminary Concordance Assessment

This study was reviewed and approved by the Ethics Committee of Hanshan Normal University, Chaozhou, China, approval No. 2024000804. Dengzhou Maternal and Child Health Hospital, Henan Province, China, served as the clinical sample collection site and provided institutional authorization for participant recruitment and blood sample collection under the approved study protocol. Written informed consent was obtained from all participants or, where applicable, from their legal guardians before sample collection. All procedures involving human‐derived samples were conducted in accordance with the Declaration of Helsinki. Peripheral blood samples were collected at Dengzhou Maternal and Child Health Hospital between May and October 2024, after ethics approval and institutional authorization had been obtained. For each participant, 3–5 mL of peripheral blood was collected, and aliquots of whole blood were applied onto filter paper to prepare DBS. The DBS samples were air‐dried, vacuum‐sealed individually, labeled with coded identifiers, transported to the laboratory, and stored at −80°C until analysis. Genotyping was performed using the TaqMan‐MGB probe assay. Sanger sequencing of PCR amplicons was performed by Sangon Biotech Co. Ltd. and the results were used as the reference method for preliminary concordance assessment. The sample‐level concordance rate was calculated as the proportion of DBS samples with concordant genotype calls between the two methods. The 95% confidence interval was calculated using the two‐sided exact binomial Clopper–Pearson method. Because of the limited sample size, this assessment was intended to provide preliminary concordance data rather than to establish definitive clinical diagnostic performance.

## 3 Results

### 3.1 Design and Strategy of the TaqMan‐MGB Assay for *CYP2C19* Polymorphism Identification

**FIGURE 1:**
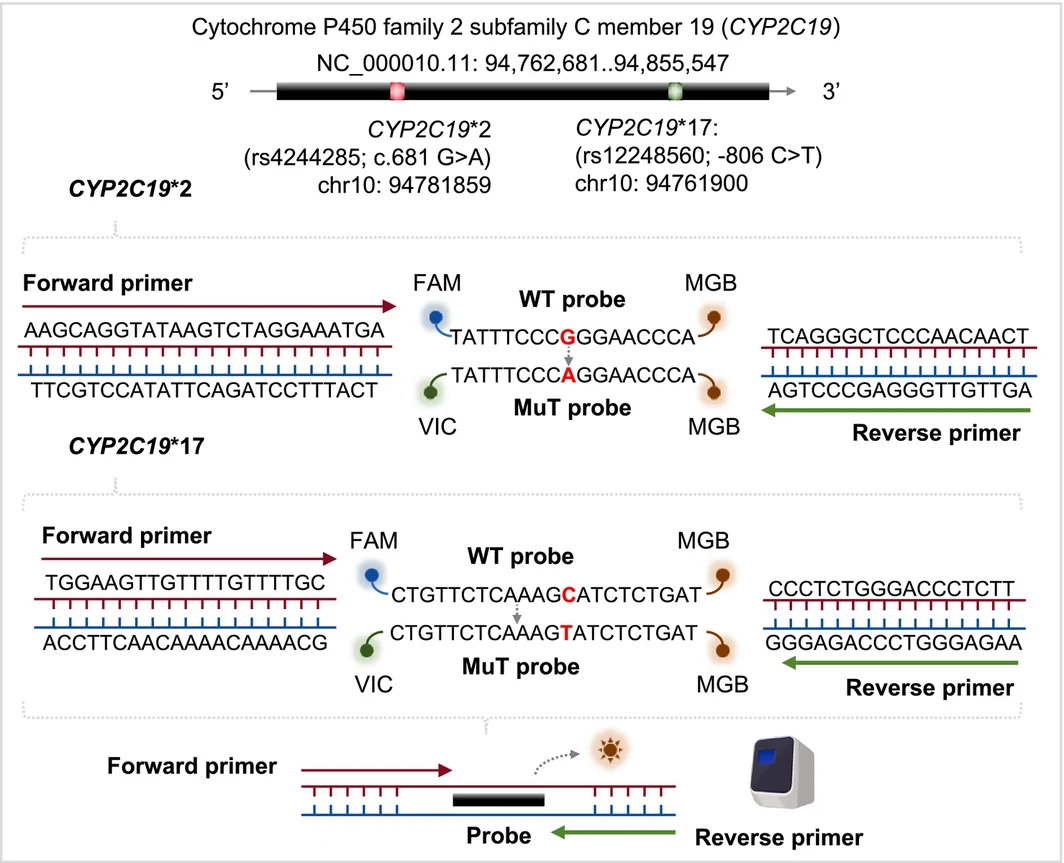
Schematic representation of the TaqMan‐MGB assay design for CYP2C19*2 and CYP2C19*17 polymorphism detection. WT, Wild type; MuT, Mutant type.

We developed a TaqMan‐MGB‐based detection platform specifically designed to identify the CYP2C19*2 (681G > A) and CYP2C19*17 (−806C > T) polymorphisms. The platform utilizes a set of highly specific primers and dual‐labeled probes to differentiate between wild‐type and mutant alleles. The probes are labeled with distinct fluorophores: FAM for the wild‐type allele and VIC for the mutant allele. To enhance specificity and stability, the probes are also conjugated with MGB groups. The fluorescence emitted during the amplification process is detected in real time by a PCR system, enabling real‐time allele discrimination. For the CYP2C19*2 polymorphism, the wild‐type probe detects the G allele, whereas the mutant probe targets the A allele. Similarly, for the CYP2C19*17 polymorphism, the wild‐type probe identifies the C allele, and the mutant probe detects the T allele. During PCR amplification, the probes bind specifically to their complementary sequences, and the Taq polymerase's 5′ → 3′ exonuclease activity degrades the probe, releasing a fluorescent signal that enables real‐time allele detection (Figure 1).

### 3.2 Optimization and Selection of Primer Combination for the TaqMan‐MGB Assay in *CYP2C19* Polymorphism Detection

**FIGURE 2:**
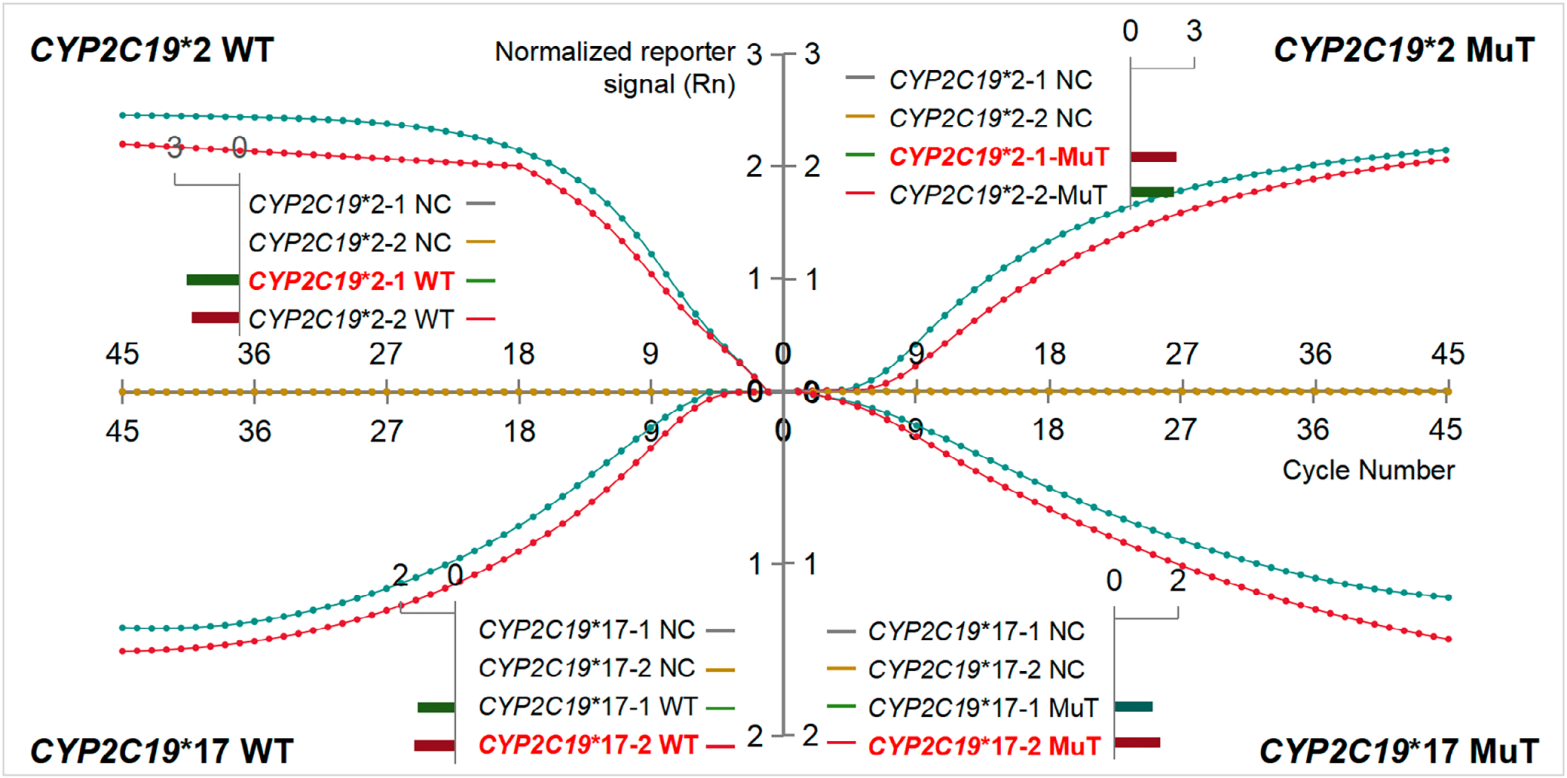
Evaluation of primer combination performance for CYP2C19*2 and CYP2C19*17 polymorphism detection via the TaqMan‐MGB assay. NC, Negative control.

To ensure the specificity and efficiency of the TaqMan‐MGB probe platform in detecting CYP2C19*2 and CYP2C19*17 polymorphisms, two primer combinations were evaluated for each polymorphism using fluorescence signal intensity as the primary criterion. Both primer combinations generated fluorescence signals in the presence of the wild‐type and mutant plasmid DNA templates, whereas the negative control remained signal‐free, indicating the absence of detectable nonspecific amplification or contamination. For the CYP2C19*2 locus, primer combination 1 presented substantially stronger fluorescence signals than did primer combination 2, indicating better amplification performance. Consequently, primer combination 1 was selected for subsequent experiments targeting CYP2C19*2. Similarly, for the CYP2C19*17 locus, primer combination 2 produced stronger fluorescence signals and was selected for downstream analyses (Figure 2).

### 3.3 Sensitivity Assessment and Candidate LODs of the TaqMan‐MGB Assay for *CYP2C19* Polymorphisms

**FIGURE 3:**
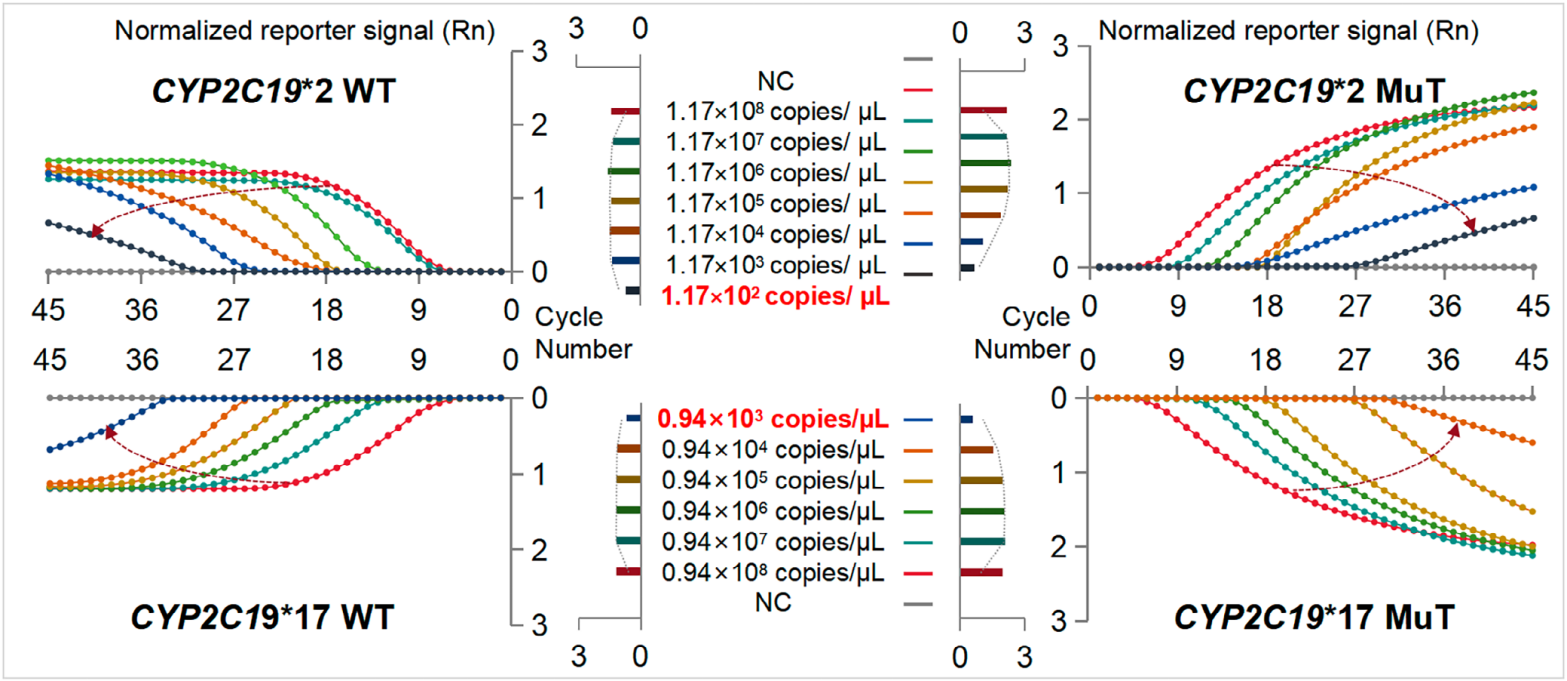
Assessment of candidate LODs for CYP2C19*2 and CYP2C19*17 via the TaqMan‐MGB assay.

The analytical sensitivity of the TaqMan‐MGB probe method for detecting *CYP2C19* polymorphisms was evaluated using gradient‐diluted standard plasmids for the CYP2C19*2 and CYP2C19*17 loci. The fluorescence signal intensity progressively decreased with decreasing plasmid concentration. In the dilution series, positive amplification was still observed at 1.17 × 10^2^ copies/μL for CYP2C19*2 and 0.94 × 10^3^ copies/μL for CYP2C19*17. These two concentrations were therefore identified as plasmid‐template candidate LODs (Figure 3). Amplification at these concentrations was further assessed in subsequent repeatability testing.

### 3.4 Specificity Evaluation of the TaqMan‐MGB Assay for Detecting *CYP2C19* Polymorphisms via Single‐ and Dual‐Probe Strategies

**FIGURE 4:**
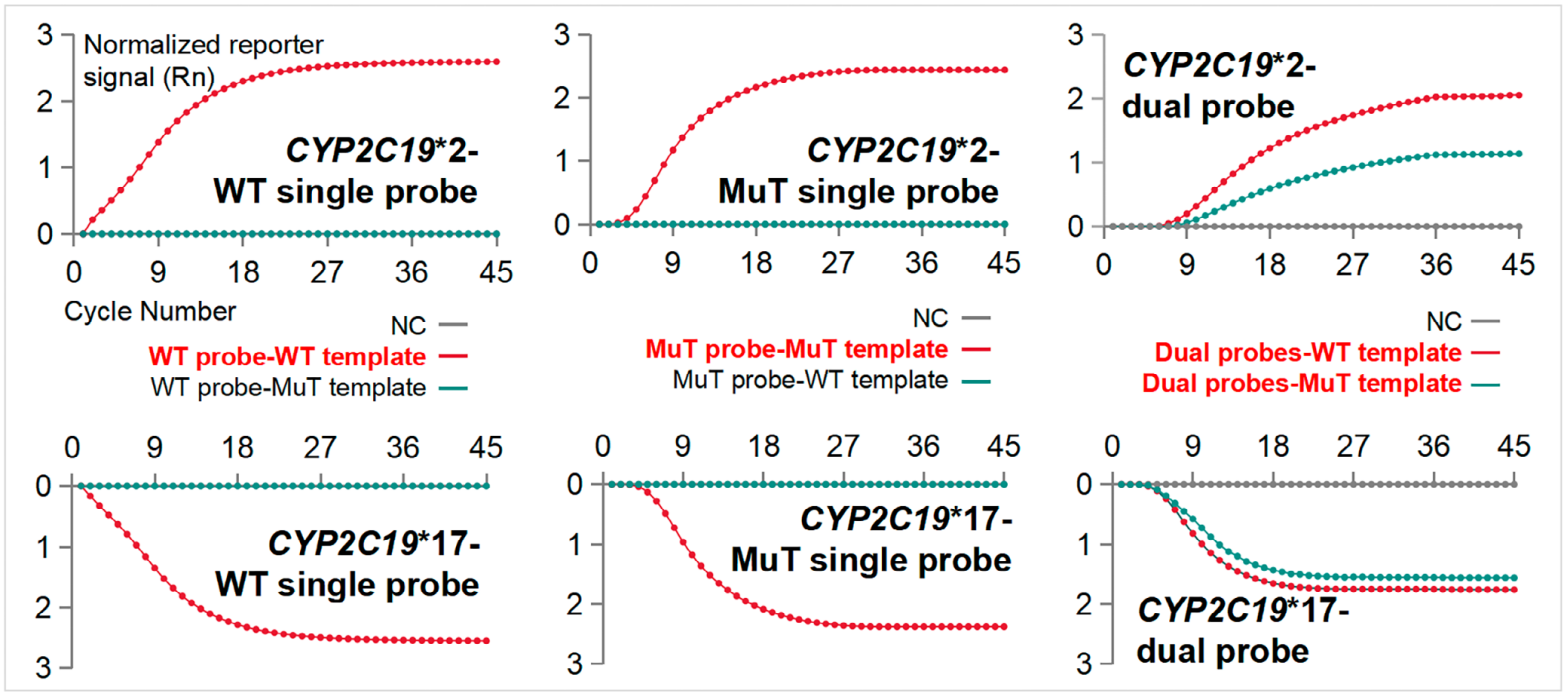
Specificity analysis of TaqMan‐MGB probes for *CYP2C19* polymorphisms via single‐ and dual‐probe approaches.

The TaqMan‐MGB probe method was employed to evaluate the specificity of detecting CYP2C19*2 and CYP2C19*17 polymorphisms via both single‐ and dual‐probe strategies. The results showed that the mutation‐specific probes generated detectable fluorescence signals with the corresponding mutant plasmid templates, whereas the wild‐type probes generated detectable fluorescence signals with the corresponding wild‐type plasmid templates. When both probes were used concurrently, each polymorphism type was accurately identified with no cross‐reactivity or interference between probes (Figure 4).

### 3.5 Repeatability Analysis of the TaqMan‐MGB Probe Method for Detecting *CYP2C19* *2 and *CYP2C19* *17 Polymorphisms

**FIGURE 5:**
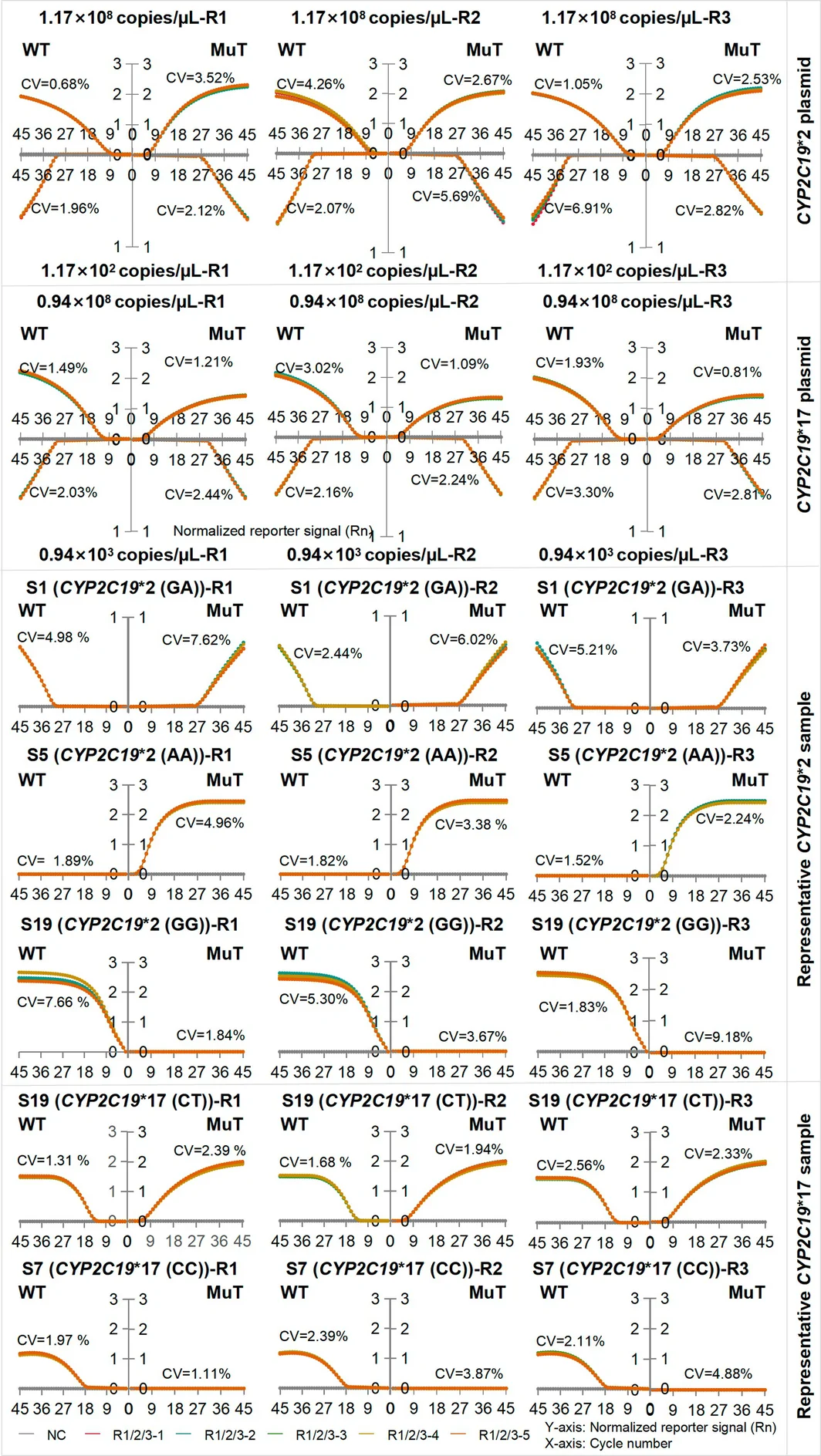
Repeatability analysis of the TaqMan‐MGB assay for CYP2C19*2 and CYP2C19*17 detection using plasmid templates and representative DBS‐derived clinical genomic DNA. CV values were calculated from endpoint ΔRn fluorescence signals recorded at cycle 45 and are indicated for each replicate set.

To evaluate the repeatability of the TaqMan‐MGB probe assay, three independent experiments were conducted under optimized conditions using both high‐concentration plasmid templates and low‐concentration templates corresponding to the candidate LODs, as well as representative DBS‐derived genomic DNA samples with Sanger‐confirmed genotypes. For CYP2C19*2, the tested concentrations were 1.17 × 10^8^ and 1.17 × 10^2^ copies/μL; for CYP2C19*17, the tested concentrations were 0.94 × 10^8^ and 0.94 × 10^3^ copies/μL. The representative clinical samples included CYP2C19*2 GA, AA and GG genotypes and CYP2C19*17 CT and CC genotypes. The CV values calculated from endpoint ΔRn fluorescence signals ranged from 0.68% to 7.66% across the plasmid‐template and DBS‐derived genomic DNA repeatability experiments, and all were below 10% (Figure 5).

### 3.6 Preliminary Concordance Assessment in Clinical DBS Samples

**FIGURE 6:**
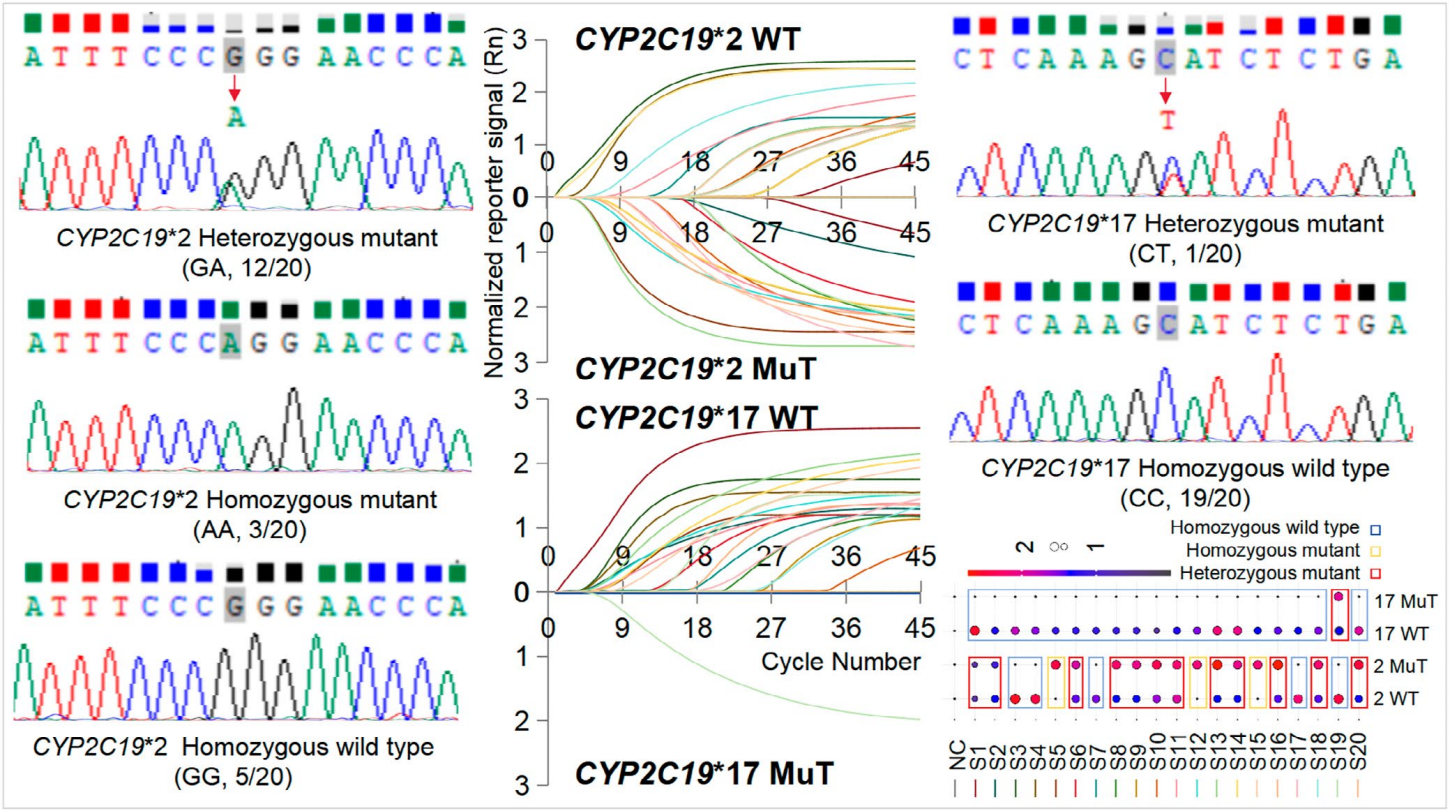
Genotyping of *CYP2C19* polymorphisms in clinical samples via the TaqMan‐MGB assay and sequencing analysis.

A total of 20 clinical DBS samples were analyzed using the TaqMan‐MGB probe assay and Sanger sequencing to preliminarily assess concordance between the two methods. For the CYP2C19*2 polymorphism, sequencing revealed the following genotype distribution: heterozygous mutant (GA) in 12 samples (60%), homozygous mutant (AA) in 3 samples (15%), and wild type (GG) in 5 samples (25%). For the CYP2C19*17 polymorphism, the observed genotypes were wild type (CC) in 19 samples (95%) and heterozygous mutant (CT) in 1 sample (5%), with no homozygous mutant TT detected. The TaqMan‐MGB assay results were concordant with Sanger sequencing for all 20 DBS samples, corresponding to an observed sample‐level concordance rate of 100% within this limited cohort and an exact binomial 95% confidence interval of 83.2%–100.0% (Figure 6).

## 4 Discussion

TaqMan‐MGB genotyping for *CYP2C19* variants is an established methodology, and commercial assays are available. Thus, the present work should be interpreted as an analytically validated, laboratory‐customized workflow integrating open primer/probe design with a DBS‐compatible Chelex‐100 extraction procedure, rather than as a fundamentally new genotyping technology. In this study, we developed and optimized a laboratory‐customized method for detecting *CYP2C19* polymorphisms at two loci, namely, CYP2C19*2 (681G > A) and CYP2C19*17 (−806C > T), via TaqMan‐MGB probes. Compared with some conventional genotyping workflows such as PCR‐RFLP or post‐PCR sequencing, TaqMan‐MGB assays can provide real‐time fluorescence‐based allele detection and may reduce post‐amplification handling. Under the tested conditions, the present assay served as an efficient workflow for the two selected *CYP2C19* variants. The incorporation of MGB groups into the probes significantly enhances their binding affinity to target sequences. This modification allows for shorter probe lengths and improves specificity, particularly in regions with high GC content or SNP‐dense loci [18]. Moreover, the method results in stable hybridization even at low DNA concentrations, effectively reducing the risk of false‐negative results [19].

The sensitivity of the TaqMan‐MGB assay is an important analytical parameter for evaluating its ability to detect *CYP2C19* polymorphisms, particularly when DNA quantity and quality vary across sample types. Using plasmid templates, positive amplification was observed at 1.17 × 10^2^ copies/μL for CYP2C19*2 and 0.94 × 10^3^ copies/μL for CYP2C19*17, and these concentrations were defined as candidate LODs under the tested conditions. However, direct comparison with other methods is limited because of differences in targets, template types, reaction systems, and LOD definitions. A previous study integrating V‐shaped PCR with CRISPR/Cas13a for *CYP2C19* genotyping reported detection at approximately 10^2^ copies/μL, although direct comparison with the present assay is limited by differences in assay design and LOD determination methods [20]. In addition to its performance in low‐concentration plasmid‐template testing, the analytical sensitivity of the TaqMan‐MGB assay indicates that further studies could explore its performance in more complex or heterogeneous sample settings. For example, in cases of chimerism or mixed populations of mutant and wild‐type alleles, the ability to amplify and detect low‐abundance targets is essential for accurate genotyping [21, 22]. Comparative studies have highlighted the favorable sensitivity and specificity of TaqMan‐MGB detection under these conditions compared with alternative methods, such as SYBR Green‐based real‐time PCR and molecular beacon assays [23, 24]. This advantage is likely attributable to the incorporation of MGB molecules, which enhance probe binding stability and improve discrimination between closely related alleles. This assay may be compatible with workflows requiring limited DNA input. However, the present study did not evaluate point‐of‐care implementation, emergency clinical decision‐making, turnaround time in real‐world settings, or clinical outcome impact. Therefore, its suitability for decentralized or rapid clinical use remains to be determined in future studies specifically designed for these settings.

The TaqMan‐MGB method demonstrates good specificity in distinguishing wild‐type and mutant alleles at the CYP2C19*2 and CYP2C19*17 loci. It accurately differentiates closely related alleles without cross‐reactivity, even when a dual‐probe strategy is employed [25]. In this study, both wild‐type and mutant probes were used simultaneously to achieve precise allele discrimination, with no interference between probes. This probe‐based strategy may reduce nonspecific signals commonly associated with some dye‐based real‐time PCR approaches, such as SYBR Green‐based real‐time PCR [26], which often suffers from reduced specificity due to nonspecific binding or primer–dimer formation. The dual‐probe strategy employed in this study further enhances detection specificity. By utilizing distinct fluorescent probes (FAM for wild‐type alleles and VIC for mutant alleles), the system effectively differentiates alleles in real‐time detection, minimizing the risk of false positive or false negative results. This dual‐probe approach has been validated in other genetic studies, such as those targeting *ABCD4* gene (Chr7:89393414, C > T) mutations [25].

The repeatability of detection methods is crucial in molecular biology experiments, especially in the clinical application of gene polymorphism analysis. Our study employed three independent experiments to assess the repeatability of the TaqMan‐MGB method, which demonstrated high consistency under identical conditions [27]. The TaqMan‐MGB probe method also exhibited excellent repeatability in Leber hereditary optic neuropathy (LHON) mtDNA (m.3460G > A, m.11778G > A, and m.14484 T > C) detection, consistently yielding high‐quality results across multiple trials [26]. Compared with other real‐time fluorescent PCR methods, the low coefficient of variation of the TaqMan‐MGB method not only confirms its repeatability but also suggests its potential for further workflow optimization. To further validate the repeatability of the TaqMan‐MGB method, experiments were conducted with varying template concentrations. The results indicated that the method maintained high accuracy and consistency regardless of whether the DNA template concentration was high or low. This performance may reduce the influence of template concentration variation within the tested range. This phenomenon is supported by evidence demonstrating that the precise probe design and real‐time data acquisition capabilities of the TaqMan‐MGB method ensure reproducible results across the broad range of DNA concentrations [28]. In response to the potential influence of clinical sample matrix effects, repeatability was further assessed using Chelex‐extracted genomic DNA from representative DBS samples. Unlike purified plasmid templates, DBS‐derived Chelex extracts may contain background human genomic DNA and residual blood‐ or resin‐associated components that can affect PCR amplification. The consistent genotype calls and CV values below 10% observed in these clinical DNA extracts provide additional support for the repeatability of the assay in a more clinically relevant sample matrix. Nevertheless, this assessment included only representative genotypes available in the current clinical sample set, and larger repeatability studies covering additional genotypes are needed.

The genotyping results from the clinical DBS samples provide preliminary support for the applicability of the TaqMan‐MGB assay in *CYP2C19* genotyping. In this limited validation set, the assay results were fully concordant with Sanger sequencing, with an observed concordance rate of 100% and an exact binomial 95% confidence interval of 83.2%–100.0%. However, the lower bound of the confidence interval and the small sample size indicate that the current data are insufficient to establish definitive analytical performance or broad clinical generalizability. Therefore, larger validation studies involving more diverse populations and a broader spectrum of *CYP2C19* genotypes are needed before the analytical robustness and potential clinical applicability of the assay can be established. This is especially critical in populations with a high prevalence of specific polymorphisms, such as the *CYP2C19* variant in East Asian populations, which necessitates treatment strategies distinct from those employed in Caucasian populations [29]. Despite the promising analytical performance observed in this study, several limitations should be acknowledged. The clinical concordance assessment was based on a relatively small sample size (*n* = 20), which is insufficient to capture the full genetic variability of *CYP2C19* across different populations. DNA concentration and purity were not systematically assessed for all DBS‐derived Chelex extracts. Chelex‐100 extraction produces crude DNA preparations that may contain resin‐associated or blood‐derived components, which can affect spectrophotometric measurements. Because the candidate LODs were determined using plasmid templates, they may not directly represent the minimum amount of DBS‐derived genomic DNA required for reliable genotyping. Future studies should include fluorometric DNA quantification, such as Qubit‐ or PicoGreen‐based assays, together with purity assessment to better evaluate the relationship among DBS input, DNA yield, and amplification performance. The assay was not directly compared with commercial TaqMan genotyping assays or non‐MGB qPCR systems; therefore, the present data do not support conclusions regarding superiority, cost‐effectiveness, or operational advantages over existing methods. Finally, this study focused only on CYP2C19*2 and CYP2C19*17 and did not include other clinically relevant alleles, such as CYP2C19*3 or rare variants. Because CYP2C19*3 is clinically important in East Asian populations including both Japanese and Chinese, this omission limits the use of the current assay as a stand‐alone test for comprehensive *CYP2C19* phenotype assignment in such populations [30]. Larger validation studies including diverse populations, broader genotype distributions, CYP2C19*3 and other additional *CYP2C19* alleles, and side‐by‐side method comparisons are warranted.

Although the study demonstrated preliminary concordance with sequencing and good repeatability under the tested conditions, larger studies involving more diverse populations are necessary to generalize the findings and assess the robustness of the method across various genetic and clinical contexts. The allele frequencies of CYP2C19*2 and CYP2C19*17 vary significantly among ethnic groups [31]. This study did not encompass the full spectrum of *CYP2C19* genetic variation, excluding clinically significant alleles such as CYP2C19*3 and rare variants [3]. This omission limits the applicability of the test in comprehensive pharmacogenomic analyses, particularly in populations with relatively high carriage rates of CYP2C19*3. Although the TaqMan‐MGB method is efficient, its reliance on specialized equipment and reagents for real‐time PCR systems may hinder accessibility in resource‐limited settings. This poses a challenge for its adoption in low‐ and middle‐income countries, which often face a greater burden of diseases requiring *CYP2C19* genotyping, such as cardiovascular conditions [32]. To address these limitations and enhance the clinical utility of TaqMan‐MGB, future iterations of the detection method should incorporate a broader range of *CYP2C19* polymorphisms. This would facilitate more comprehensive pharmacogenetic analyses, especially for populations with unique allele distributions. Large‐scale validation studies involving participants from diverse ethnic backgrounds are crucial to confirm the accuracy and repeatability of the test across different genetic contexts. Additionally, integrating TaqMan‐MGB with emerging technologies, such as microfluidics or portable PCR platforms, could improve its scalability and accessibility. Embedding the test into point‐of‐care testing devices would enable rapid genotyping in decentralized or resource‐constrained healthcare environments.

## 5 Conclusion

The TaqMan‐MGB probe assay developed in this study provides a laboratory‐customized method for detecting the CYP2C19*2 and CYP2C19*17 polymorphisms. Under the optimized experimental conditions, the assay showed good analytical specificity, sensitivity, and repeatability, and its genotyping results were concordant with Sanger sequencing in a small set of clinical DBS samples. However, because the clinical sample size was limited, the CYP2C19*17 TT genotype was not represented, and no direct comparison with commercial TaqMan assays or non‐MGB qPCR methods was performed; further validation is required before its relative performance, clinical robustness, and generalizability can be established. Future studies should expand the sample size, include additional clinically important *CYP2C19* alleles, and perform side‐by‐side comparisons with existing genotyping platforms across diverse populations.

## Author Contributions

Yaqun Liu: Conceptualization, Methodology, Investigation, Writing – original draft. Lianghui Chen: Software, Validation. Xuanyi Zheng: Resources, Data curation. Peikui Yang: Data curation, Writing – review and editing. Yicun Chen: Supervision, Project administration. Chengsong Xie: Funding acquisition, Project administration. Zhenxia Zhang: Data curation, Formal analysis, Visualization. Yuzhong Zheng: Conceptualization, Methodology, Supervision, Writing – review and editing, Funding acquisition.

## Funding

This work was supported by Guangdong Key Laboratory of Functional Substances and Health Products from Medicinal Edible Resources, 2021B1212040015. Guangdong Provincial Department of Education Project, 2025KQNCX043. Hanshan Normal University Quality Engineering Program, HSJG‐SZ252187. Chaozhou Science and Technology Bureau Science and Technology Project, 202404GY06.

## Conflicts of Interest

The authors declare no conflicts of interest.

## Data Availability

The data that support the findings of this study are available from the corresponding authors upon reasonable request. Individual‐level clinical genetic data are not publicly available due to ethical and privacy restrictions. Relevant primer/probe sequences and summarized validation data are provided within the article.
